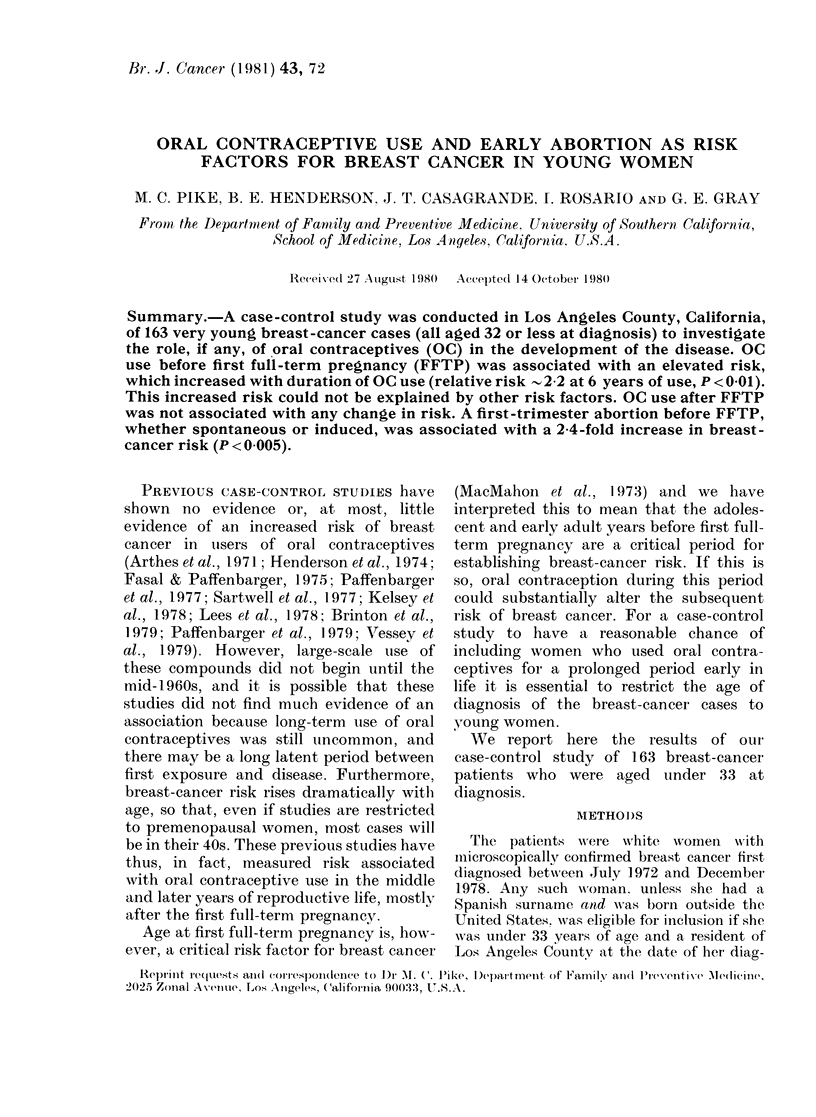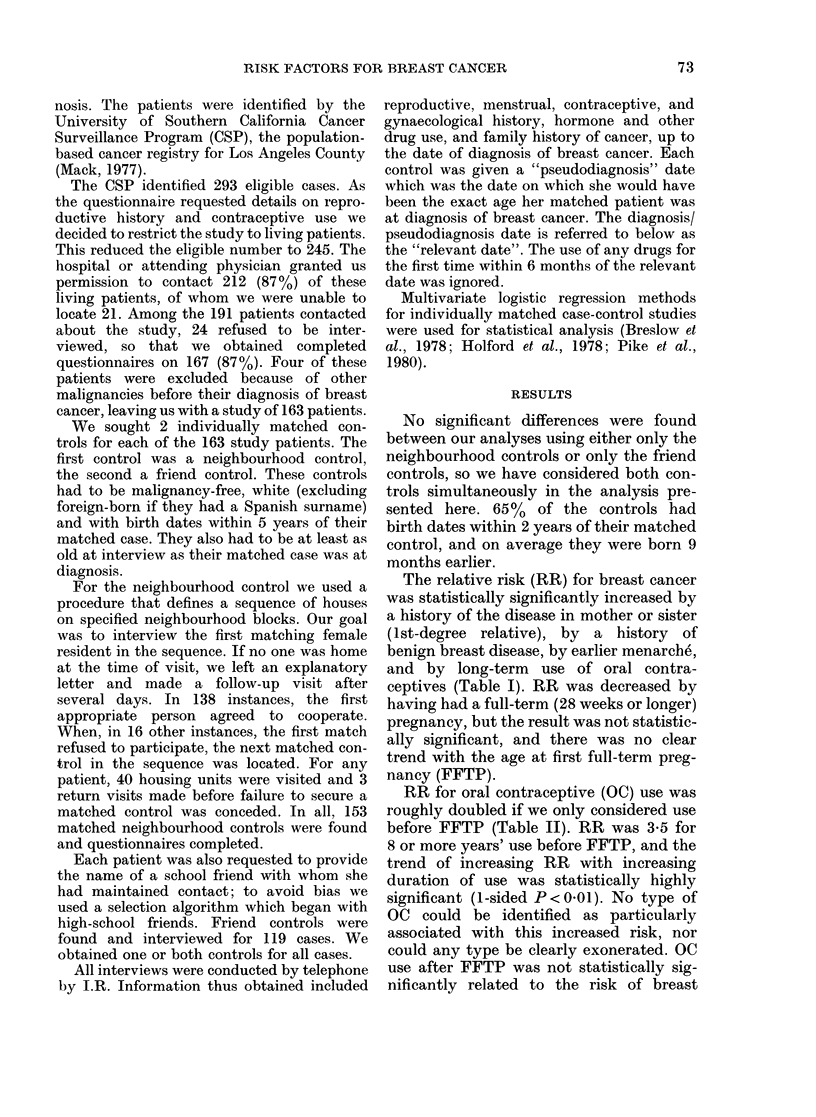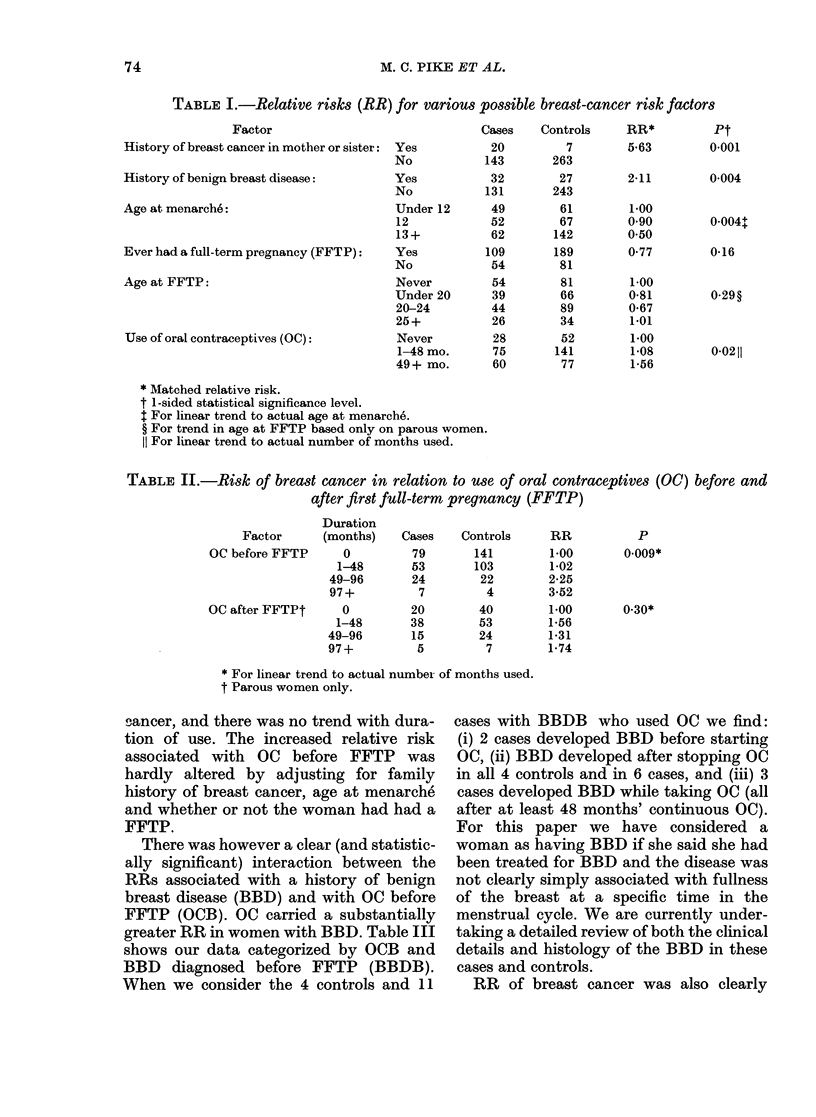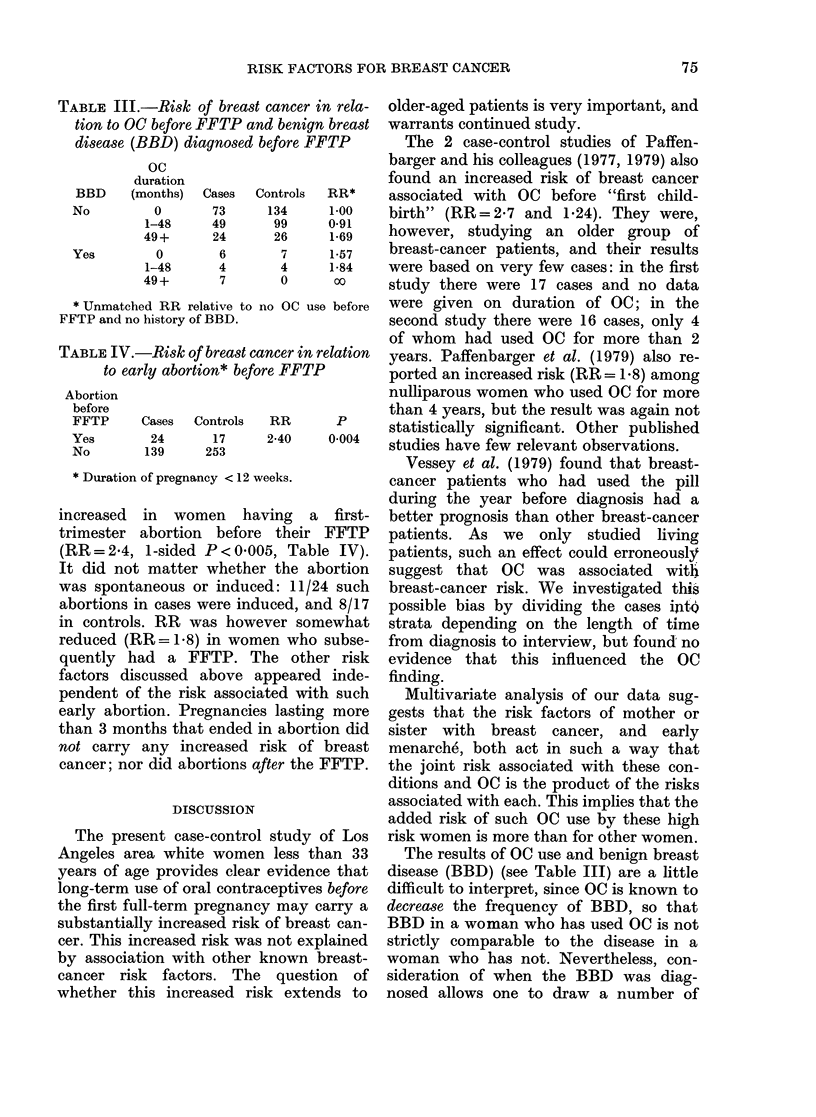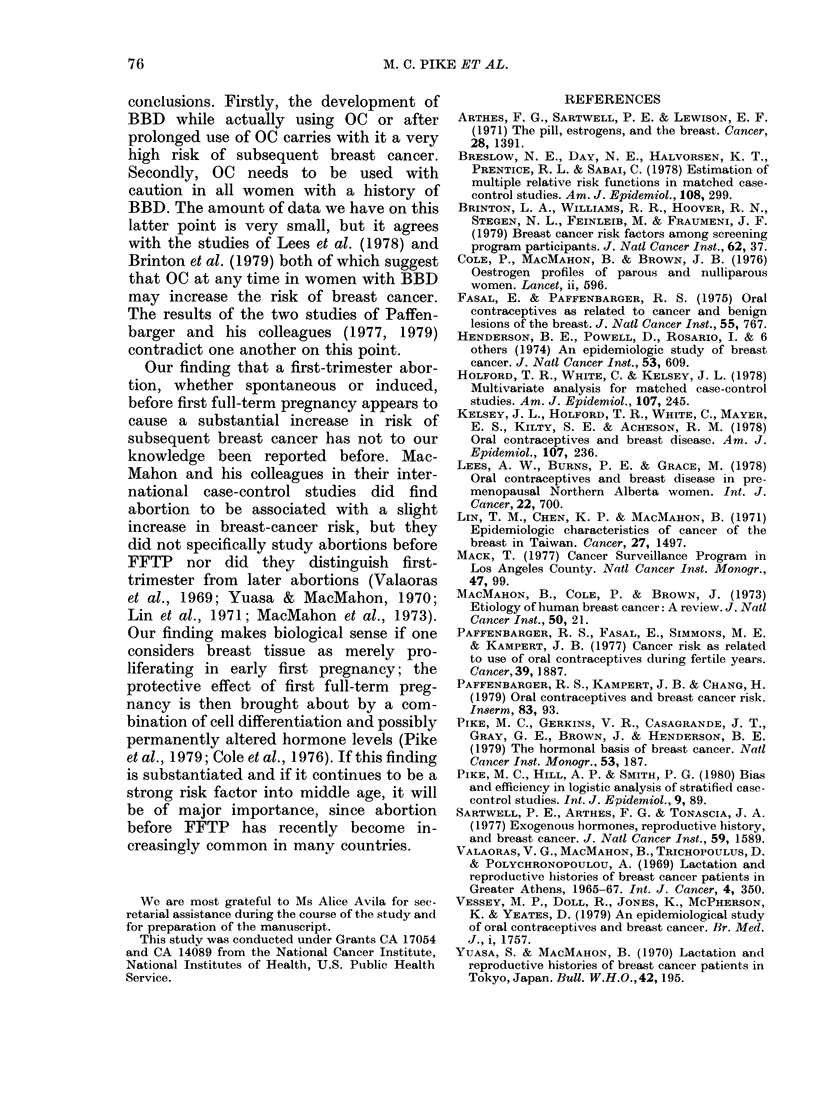# Oral contraceptive use and early abortion as risk factors for breast cancer in young women.

**DOI:** 10.1038/bjc.1981.10

**Published:** 1981-01

**Authors:** M. C. Pike, B. E. Henderson, J. T. Casagrande, I. Rosario, G. E. Gray

## Abstract

A case-control study was conducted in Los Angeles County, California, of 163 very young breast-cancer cases (all aged 32 or less at diagnosis) to investigate the role, if any, of oral contraceptives (OC) in the development of the disease. OC use before first full-term pregnancy (FFTP) was associated with an elevated risk, which increased with duration of OC use (relative risk approximately 2.2 at 6 years of use, P < 0.01). This increased risk could not be explained by other risk factors. OC use after FFTP was not associated with any change in risk. A first-trimester abortion before FFTP, whether spontaneous or induced, was associated with a 2.4-fold increase in breast-cancer risk (P < 0.005).


*Br. J. Cancer* (1981) **43,** 72

### ORAL CONTRACEPTIVE USE AND EARLY ABORTION AS RISK FACTORS FOR BREAST CANCER IN YOUNG WOMEN

M. C. PIKE, B. E. HENDERSON, J. T. CASAGRANDE, I. ROSARIO AND G. E. GRAY

*From the Department of Family and Preventive Medicine, University of Southern California, School of Medicine, Los Angeles, California, U.S.A.*

Received 27 August 1980 Accepted 14 October 1980

**Summary.—A case-control study was conducted in Los Angeles County, California, of 163 very young breast-cancer cases (all aged 32 or less at diagnosis) to investigate the role, if any, of oral contraceptives (OC) in the development of the disease. OC use before first full-term pregnancy (FFTP) was associated with an elevated risk, which increased with duration of OC use (relative risk ~2·2 at 6 years of use, $P<0{\cdot}01$). This increased risk could not be explained by other risk factors. OC use after FFTP was not associated with any change in risk. A first-trimester abortion before FFTP, whether spontaneous or induced, was associated with a 2·4-fold increase in breast-cancer risk ($P<0{\cdot}005$).**

PREVIOUS CASE-CONTROL STUDIES have shown no evidence or, at most, little evidence of an increased risk of breast cancer in users of oral contraceptives (Arthes *et al.*, 1971; Henderson *et al.*, 1974; Fasal & Paffenbarger, 1975; Paffenbarger *et al.*, 1977; Sartwell *et al.*, 1977; Kelsey *et al.*, 1978; Lees *et al.*, 1978; Brinton *et al.*, 1979; Paffenbarger *et al.*, 1979; Vessey *et al.*, 1979). However, large-scale use of these compounds did not begin until the mid-1960s, and it is possible that these studies did not find much evidence of an association because long-term use of oral contraceptives was still uncommon, and there may be a long latent period between first exposure and disease. Furthermore, breast-cancer risk rises dramatically with age, so that, even if studies are restricted to premenopausal women, most cases will be in their 40s. These previous studies have thus, in fact, measured risk associated with oral contraceptive use in the middle and later years of reproductive life, mostly after the first full-term pregnancy.

Age at first full-term pregnancy is, however, a critical risk factor for breast cancer (MacMahon *et al.*, 1973) and we have interpreted this to mean that the adolescent and early adult years before first full-term pregnancy are a critical period for establishing breast-cancer risk. If this is so, oral contraception during this period could substantially alter the subsequent risk of breast cancer. For a case-control study to have a reasonable chance of including women who used oral contraceptives for a prolonged period early in life it is essential to restrict the age of diagnosis of the breast-cancer cases to young women.

We report here the results of our case-control study of 163 breast-cancer patients who were aged under 33 at diagnosis.

### METHODS

The patients were white women with microscopically confirmed breast cancer first diagnosed between July 1972 and December 1978. Any such woman, unless she had a Spanish surname *and* was born outside the United States, was eligible for inclusion if she was under 33 years of age and a resident of Los Angeles County at the date of her diag-

Reprint requests and correspondence to Dr M. C. Pike, Department of Family and Preventive Medicine, 2025 Zonal Avenue, Los Angeles, California 90033, U.S.A.

nosis. The patients were identified by the University of Southern California Cancer Surveillance Program (CSP), the population-based cancer registry for Los Angeles County (Mack, 1977).

The CSP identified 293 eligible cases. As the questionnaire requested details on reproductive history and contraceptive use we decided to restrict the study to living patients. This reduced the eligible number to 245. The hospital or attending physician granted us permission to contact 212 (87%) of these living patients, of whom we were unable to locate 21. Among the 191 patients contacted about the study, 24 refused to be interviewed, so that we obtained completed questionnaires on 167 (87%). Four of these patients were excluded because of other malignancies before their diagnosis of breast cancer, leaving us with a study of 163 patients.

We sought 2 individually matched controls for each of the 163 study patients. The first control was a neighbourhood control, the second a friend control. These controls had to be malignancy-free, white (excluding foreign-born if they had a Spanish surname) and with birth dates within 5 years of their matched case. They also had to be at least as old at interview as their matched case was at diagnosis.

For the neighbourhood control we used a procedure that defines a sequence of houses on specified neighbourhood blocks. Our goal was to interview the first matching female resident in the sequence. If no one was home at the time of visit, we left an explanatory letter and made a follow-up visit after several days. In 138 instances, the first appropriate person agreed to cooperate. When, in 16 other instances, the first match refused to participate, the next matched control in the sequence was located. For any patient, 40 housing units were visited and 3 return visits made before failure to secure a matched control was conceded. In all, 153 matched neighbourhood controls were found and questionnaires completed.

Each patient was also requested to provide the name of a school friend with whom she had maintained contact; to avoid bias we used a selection algorithm which began with high-school friends. Friend controls were found and interviewed for 119 cases. We obtained one or both controls for all cases.

All interviews were conducted by telephone by I.R. Information thus obtained included reproductive, menstrual, contraceptive, and gynaecological history, hormone and other drug use, and family history of cancer, up to the date of diagnosis of breast cancer. Each control was given a "pseudodiagnosis" date which was the date on which she would have been the exact age her matched patient was at diagnosis of breast cancer. The diagnosis/pseudodiagnosis date is referred to below as the "relevant date". The use of any drugs for the first time within 6 months of the relevant date was ignored.

Multivariate logistic regression methods for individually matched case-control studies were used for statistical analysis (Breslow *et al.*, 1978; Holford *et al.*, 1978; Pike *et al.*, 1980).

### RESULTS

No significant differences were found between our analyses using either only the neighbourhood controls or only the friend controls, so we have considered both controls simultaneously in the analysis presented here. 65% of the controls had birth dates within 2 years of their matched control, and on average they were born 9 months earlier.

The relative risk (RR) for breast cancer was statistically significantly increased by a history of the disease in mother or sister (1st-degree relative), by a history of benign breast disease, by earlier menarché, and by long-term use of oral contraceptives (Table I). RR was decreased by having had a full-term (28 weeks or longer) pregnancy, but the result was not statistically significant, and there was no clear trend with the age at first full-term pregnancy (FFTP).

RR for oral contraceptive (OC) use was roughly doubled if we only considered use before FFTP (Table II). RR was 3·5 for 8 or more years' use before FFTP, and the trend of increasing RR with increasing duration of use was statistically highly significant (1-sided $P < 0{\cdot}01$). No type of OC could be identified as particularly associated with this increased risk, nor could any type be clearly exonerated. OC use after FFTP was not statistically significantly related to the risk of breast

TABLE I.—*Relative risks (RR) for various possible breast-cancer risk factors*

| Factor | | Cases | Controls | RR* | P† |
|---|---|---|---|---|---|
| History of breast cancer in mother or sister: | Yes | 20 | 7 | 5·63 | 0·001 |
| | No | 143 | 263 | | |
| History of benign breast disease: | Yes | 32 | 27 | 2·11 | 0·004 |
| | No | 131 | 243 | | |
| Age at menarché: | Under 12 | 49 | 61 | 1·00 | |
| | 12 | 52 | 67 | 0·90 | 0·004‡ |
| | 13+ | 62 | 142 | 0·50 | |
| Ever had a full-term pregnancy (FFTP): | Yes | 109 | 189 | 0·77 | 0·16 |
| | No | 54 | 81 | | |
| Age at FFTP: | Never | 54 | 81 | 1·00 | |
| | Under 20 | 39 | 66 | 0·81 | 0·29§ |
| | 20–24 | 44 | 89 | 0·67 | |
| | 25+ | 26 | 34 | 1·01 | |
| Use of oral contraceptives (OC): | Never | 28 | 52 | 1·00 | |
| | 1–48 mo. | 75 | 141 | 1·08 | 0·02‖ |
| | 49+ mo. | 60 | 77 | 1·56 | |

* Matched relative risk.
† 1-sided statistical significance level.
‡ For linear trend to actual age at menarché.
§ For trend in age at FFTP based only on parous women.
‖ For linear trend to actual number of months used.

TABLE II.—*Risk of breast cancer in relation to use of oral contraceptives (OC) before and after first full-term pregnancy (FFTP)*

| Factor | Duration (months) | Cases | Controls | RR | *P* |
|---|---|---|---|---|---|
| OC before FFTP | 0 | 79 | 141 | 1·00 | 0·009* |
| | 1–48 | 53 | 103 | 1·02 | |
| | 49–96 | 24 | 22 | 2·25 | |
| | 97+ | 7 | 4 | 3·52 | |
| OC after FFTP† | 0 | 20 | 40 | 1·00 | 0·30* |
| | 1–48 | 38 | 53 | 1·56 | |
| | 49–96 | 15 | 24 | 1·31 | |
| | 97+ | 5 | 7 | 1·74 | |

* For linear trend to actual number of months used.
† Parous women only.

cancer, and there was no trend with duration of use. The increased relative risk associated with OC before FFTP was hardly altered by adjusting for family history of breast cancer, age at menarché and whether or not the woman had had a FFTP.

There was however a clear (and statistically significant) interaction between the RRs associated with a history of benign breast disease (BBD) and with OC before FFTP (OCB). OC carried a substantially greater RR in women with BBD. Table III shows our data categorized by OCB and BBD diagnosed before FFTP (BBDB). When we consider the 4 controls and 11 cases with BBDB who used OC we find: (i) 2 cases developed BBD before starting OC, (ii) BBD developed after stopping OC in all 4 controls and in 6 cases, and (iii) 3 cases developed BBD while taking OC (all after at least 48 months' continuous OC). For this paper we have considered a woman as having BBD if she said she had been treated for BBD and the disease was not clearly simply associated with fullness of the breast at a specific time in the menstrual cycle. We are currently undertaking a detailed review of both the clinical details and histology of the BBD in these cases and controls.

RR of breast cancer was also clearly

TABLE III.—*Risk of breast cancer in relation to OC before FFTP and benign breast disease (BBD) diagnosed before FFTP*

| BBD | OC duration (months) | Cases | Controls | RR* |
|---|---|---|---|---|
| No | 0 | 73 | 134 | 1·00 |
| | 1–48 | 49 | 99 | 0·91 |
| | 49+ | 24 | 26 | 1·69 |
| Yes | 0 | 6 | 7 | 1·57 |
| | 1–48 | 4 | 4 | 1·84 |
| | 49+ | 7 | 0 | ∞ |

* Unmatched RR relative to no OC use before FFTP and no history of BBD.

TABLE IV.—*Risk of breast cancer in relation to early abortion* before FFTP*

| Abortion before FFTP | Cases | Controls | RR | $P$ |
|---|---|---|---|---|
| Yes | 24 | 17 | 2·40 | 0·004 |
| No | 139 | 253 | | |

* Duration of pregnancy < 12 weeks.

increased in women having a first-trimester abortion before their FFTP (RR = 2·4, 1-sided $P < 0{\cdot}005$, Table IV). It did not matter whether the abortion was spontaneous or induced: 11/24 such abortions in cases were induced, and 8/17 in controls. RR was however somewhat reduced (RR = 1·8) in women who subsequently had a FFTP. The other risk factors discussed above appeared independent of the risk associated with such early abortion. Pregnancies lasting more than 3 months that ended in abortion did *not* carry any increased risk of breast cancer; nor did abortions *after* the FFTP.

### DISCUSSION

The present case-control study of Los Angeles area white women less than 33 years of age provides clear evidence that long-term use of oral contraceptives *before* the first full-term pregnancy may carry a substantially increased risk of breast cancer. This increased risk was not explained by association with other known breast-cancer risk factors. The question of whether this increased risk extends to older-aged patients is very important, and warrants continued study.

The 2 case-control studies of Paffenbarger and his colleagues (1977, 1979) also found an increased risk of breast cancer associated with OC before "first childbirth" (RR = 2·7 and 1·24). They were, however, studying an older group of breast-cancer patients, and their results were based on very few cases: in the first study there were 17 cases and no data were given on duration of OC; in the second study there were 16 cases, only 4 of whom had used OC for more than 2 years. Paffenbarger *et al.* (1979) also reported an increased risk (RR = 1·8) among nulliparous women who used OC for more than 4 years, but the result was again not statistically significant. Other published studies have few relevant observations.

Vessey *et al.* (1979) found that breast-cancer patients who had used the pill during the year before diagnosis had a better prognosis than other breast-cancer patients. As we only studied living patients, such an effect could erroneously suggest that OC was associated with breast-cancer risk. We investigated this possible bias by dividing the cases into strata depending on the length of time from diagnosis to interview, but found no evidence that this influenced the OC finding.

Multivariate analysis of our data suggests that the risk factors of mother or sister with breast cancer, and early menarché, both act in such a way that the joint risk associated with these conditions and OC is the product of the risks associated with each. This implies that the added risk of such OC use by these high risk women is more than for other women.

The results of OC use and benign breast disease (BBD) (see Table III) are a little difficult to interpret, since OC is known to *decrease* the frequency of BBD, so that BBD in a woman who has used OC is not strictly comparable to the disease in a woman who has not. Nevertheless, consideration of when the BBD was diagnosed allows one to draw a number of conclusions. Firstly, the development of BBD while actually using OC or after prolonged use of OC carries with it a very high risk of subsequent breast cancer. Secondly, OC needs to be used with caution in all women with a history of BBD. The amount of data we have on this latter point is very small, but it agrees with the studies of Lees *et al.* (1978) and Brinton *et al.* (1979) both of which suggest that OC at any time in women with BBD may increase the risk of breast cancer. The results of the two studies of Paffenbarger and his colleagues (1977, 1979) contradict one another on this point.

Our finding that a first-trimester abortion, whether spontaneous or induced, before first full-term pregnancy appears to cause a substantial increase in risk of subsequent breast cancer has not to our knowledge been reported before. MacMahon and his colleagues in their international case-control studies did find abortion to be associated with a slight increase in breast-cancer risk, but they did not specifically study abortions before FFTP nor did they distinguish first-trimester from later abortions (Valaoras *et al.*, 1969; Yuasa & MacMahon, 1970; Lin *et al.*, 1971; MacMahon *et al.*, 1973). Our finding makes biological sense if one considers breast tissue as merely proliferating in early first pregnancy; the protective effect of first full-term pregnancy is then brought about by a combination of cell differentiation and possibly permanently altered hormone levels (Pike *et al.*, 1979; Cole *et al.*, 1976). If this finding is substantiated and if it continues to be a strong risk factor into middle age, it will be of major importance, since abortion before FFTP has recently become increasingly common in many countries.

We are most grateful to Ms Alice Avila for secretarial assistance during the course of the study and for preparation of the manuscript.

This study was conducted under Grants CA 17054 and CA 14089 from the National Cancer Institute, National Institutes of Health, U.S. Public Health Service.

### REFERENCES

Arthes, F. G., Sartwell, P. E. & Lewison, E. F. (1971) The pill, estrogens, and the breast. *Cancer*, **28**, 1391.

Breslow, N. E., Day, N. E., Halvorsen, K. T., Prentice, R. L. & Sabai, C. (1978) Estimation of multiple relative risk functions in matched case-control studies. *Am. J. Epidemiol.*, **108**, 299.

Brinton, L. A., Williams, R. R., Hoover, R. N., Stegen, N. L., Feinleib, M. & Fraumeni, J. F. (1979) Breast cancer risk factors among screening program participants. *J. Natl Cancer Inst.*, **62**, 37.

Cole, P., MacMahon, B. & Brown, J. B. (1976) Oestrogen profiles of parous and nulliparous women. *Lancet*, ii, 596.

Fasal, E. & Paffenbarger, R. S. (1975) Oral contraceptives as related to cancer and benign lesions of the breast. *J. Natl Cancer Inst.*, **55**, 767.

Henderson, B. E., Powell, D., Rosario, I. & 6 others (1974) An epidemiologic study of breast cancer. *J. Natl Cancer Inst.*, **53**, 609.

Holford, T. R., White, C. & Kelsey, J. L. (1978) Multivariate analysis for matched case-control studies. *Am. J. Epidemiol.*, **107**, 245.

Kelsey, J. L., Holford, T. R., White, C., Mayer, E. S., Kilty, S. E. & Acheson, R. M. (1978) Oral contraceptives and breast disease. *Am. J. Epidemiol.*, **107**, 236.

Lees, A. W., Burns, P. E. & Grace, M. (1978) Oral contraceptives and breast disease in premenopausal Northern Alberta women. *Int. J. Cancer*, **22**, 700.

Lin, T. M., Chen, K. P. & MacMahon, B. (1971) Epidemiologic characteristics of cancer of the breast in Taiwan. *Cancer*, **27**, 1497.

Mack, T. (1977) Cancer Surveillance Program in Los Angeles County. *Natl Cancer Inst. Monogr.*, **47**, 99.

MacMahon, B., Cole, P. & Brown, J. (1973) Etiology of human breast cancer: A review. *J. Natl Cancer Inst.*, **50**, 21.

Paffenbarger, R. S., Fasal, E., Simmons, M. E. & Kampert, J. B. (1977) Cancer risk as related to use of oral contraceptives during fertile years. *Cancer*, **39**, 1887.

Paffenbarger, R. S., Kampert, J. B. & Chang, H. (1979) Oral contraceptives and breast cancer risk. *Inserm*, **83**, 93.

Pike, M. C., Gerkins, V. R., Casagrande, J. T., Gray, G. E., Brown, J. & Henderson, B. E. (1979) The hormonal basis of breast cancer. *Natl Cancer Inst. Monogr.*, **53**, 187.

Pike, M. C., Hill, A. P. & Smith, P. G. (1980) Bias and efficiency in logistic analysis of stratified case-control studies. *Int. J. Epidemiol.*, **9**, 89.

Sartwell, P. E., Arthes, F. G. & Tonascia, J. A. (1977) Exogenous hormones, reproductive history, and breast cancer. *J. Natl Cancer Inst.*, **59**, 1589.

Valaoras, V. G., MacMahon, B., Trichopoulus, D. & Polychronopoulou, A. (1969) Lactation and reproductive histories of breast cancer patients in Greater Athens, 1965–67. *Int. J. Cancer*, **4**, 350.

Vessey, M. P., Doll, R., Jones, K., McPherson, K. & Yeates, D. (1979) An epidemiological study of oral contraceptives and breast cancer. *Br. Med. J.*, i, 1757.

Yuasa, S. & MacMahon, B. (1970) Lactation and reproductive histories of breast cancer patients in Tokyo, Japan. *Bull. W.H.O.*, **42**, 195.